# Partial Substitution of Fish Meal with Soy Protein Concentrate on Growth, Liver Health, Intestinal Morphology, and Microbiota in Juvenile Large Yellow Croaker (*Larimichthys crocea*)

**DOI:** 10.1155/2023/3706709

**Published:** 2023-01-06

**Authors:** Xuexi Wang, Hongjie Luo, Dejuan Wang, Yunzong Zheng, Wenbo Zhu, Weini Zhang, Zhengbang Chen, Xinhua Chen, Jianchun Shao

**Affiliations:** ^1^Key Laboratory of Marine Biotechnology of Fujian Province, College of Marine Sciences, Fujian Agriculture and Forestry University, Fuzhou 350002, China; ^2^Fuzhou Haima Feed Co., Ltd., Fuzhou 350002, China

## Abstract

The present study investigated the growth performance, feed utilization, intestinal morphology, and microbiota communities of juvenile large yellow croaker (*Larimichthys crocea*) fed diets containing different proportions of soy protein concentrate (SPC) (0, 15%, 30%, and 45%, namely FM, SPC15, SPC30, and SPC45) as a substitute for fish meal (FM) for 8 weeks. The weight gain (WG) and specific growth rate (SGR) in fish fed SPC45 were significantly lower than those fed FM and SPC15 but not differ with these fed SPC30. The feed efficiency (FE) and protein efficiency ratio (PER) decreased sharply when the dietary SPC inclusion level was higher than 15%. The activity of alanine aminotransferase (ALT) and expression of *alt* and aspartate aminotransferase (*ast*) were significantly higher in fish fed SPC45 than those fed FM. The activity and mRNA expression of acid phosphatase were opposite. The villi height (VH) in distal intestine (DI) showed a significant quadratic response to increasing dietary SPC inclusion levels and was highest in SPC15. The VH in proximal intestine, middle intestine decreased significantly with increasing dietary SPC levels. The 16S rRNA sequences in intestine revealed that fish fed SPC15 had higher bacterial diversity and abundance of Phylum *Firmicutes* such as order *Lactobacillales* and order *Rhizobiaceae* than those fed other diets. Genus *vibrio*, family *Vibrionaceae* and order *Vibrionales* within phylum *Proteobacteria* were enriched in fish fed FM and SPC30 diets. *Tyzzerella* and *Shewanella* that belongs to phylum *Firmicutes* and *Proteobacteria,* respectively, were enriched in fish fed SPC45 diet. Our results indicated that SPC replacing more than 30% FM could lead to lower quality diet, retard growth performance, ill health, disordered intestine structure, and microbiota communities. *Tyzzerella* could be the bacteria indicator of intestinal in large yellow croaker fed low quality diet due to high SPC content. Based on the quadratic regression analysis of WG, the best growth performance could be observed when the replacement of FM with SPC was 9.75%.

## 1. Introduction

Carnivorous fish aquaculture is currently supported by the production of balanced diets, the primary components of which are animal-derived elements [1]. Fish meal (FM) is one of the most important and widely utilized protein sources in aquatic animal feed because of its high-protein quality, generally balanced fatty acids and amino acids profile, abundant vitamins and minerals, and the exceptional palatability. However, as FM is declining due to the overexploitation of pelagic fish and the rapidly rising aquaculture industry, the quest for alternate protein sources is increasingly necessary [2].

Plant proteins have been and continue to be the main protein sources due to their relatively low price and enormous production. The anti-nutritional factors (ANFs) in plant feedstuffs, on the other hand, can impair feed intake, nutrient digestibility, and utilization, as well as modify disease resistance, thus resulting in poor fish growth and development [3–5]. The presence of ANFs is widely acknowledged as one of the major drawbacks of soybean meal (SBM), limiting its inclusion level in aquatic animal diets [6, 7]. In this regard, some researchers have focused their attention on soy protein concentrate (SPC) [8–10], which is produced by extracting defatted soy flakes in aqueous ethanol or methanol to decrease or eliminate ANFs contents [11]. Moreover, SPC have several benefits over SBM, such as a favorable amino acids profile, digestible protein and energy and better palatability [12], whereas relatively lower lysine and methionine contents than FM. Previous study in large yellow croaker (*Larimichthys crocea*) demonstrates that SPC combined with lysine and methionine could totally replace dietary FM [13]. Given that the potential of SPC is worth to be studied and further clarified. Additionally, the intestine of fish is a complex microbial ecosystem that houses a dynamic consortium of microorganisms related with diet composition and can perform pivotal roles in host health, including nutrition absorption, energy generation, and immune response balance [14, 15]. To the best of our knowledge, there is no study about evaluating the using of SPC without crystal amino acid supplementation in the diet of large yellow croaker (*Larimichthys crocea*) and its subsequent effects on the fish's intestine so far.

*L. crocea* is an economically important carnivorous marine fish species in south China because of its excellent taste and important commercial value. The yield of farmed large yellow croaker was 254224 tons according to the China Fishery Statistical Yearbook [16]. Over the past years, researchers have investigated the effects of dietary FM replacement with SBM, fermented soybean meal, corn-gluten meal, meat and bone meal, and Antarctic krill meal on growth performance, tissues composition and intestinal morphology and microbiota in *L. crocea* [17–21]. These studies demonstrated the possibility of using alternative protein sources in the diets of large yellow croaker. Therefore, this study is aimed at evaluating the effects of replacing FM in the diet by SPC (without crystal amino acid supplementation), as assessed by growth performance, feed utilization, intestinal morphology, and microbiota populations in *L. crocea*.

## 2. Materials and Methods

### 2.1. Diets, Feeding Trial and Sampling

Five isonitrogenous and isolipidic (45% and 10% crude protein and lipid, respectively) were formulated to meet the requirement of large yellow croaker [13]. The dietary fish meal was replaced by SPC at: 0, 15%, 30%, and 75%, and named FM, SPC15, SPC30, and SPC45, respectively (Table 1). The amino acid profiles of the experimental diets are shown in Table 2. The experiment diets with uniform pellet size (2.0 × 5.0 mm) were manufactured as described previously [22], after which the diets were stored in a refrigerator at -20°C.

Juvenile fish were obtained from a local commercial farm (Ningde, Fujian, China), and were acclimated in floating cages (4.0 × 2.0 × 2.5 m, length × width × depth) for 2 weeks prior to the experiment. A total of 960 juveniles (19.50 ± 1.62 g) were randomly allocated into 16 floating cages (1.0 × 1.0 × 1.5 m, length × width × depth), and four replicates (60 fish per replicates) were assigned to each treatment. Fish were hand-fed to apparent satiation twice daily (5 : 30 and 17 : 30) for 8 weeks. During the period of the experiment, the water temperature, dissolved oxygen level and ammonia nitrogen level were 28.0 ± 0.8°C, 6.5 ± 0.7 mg L^−1^, and 0.20 ± 0.04 mg L^−1^, respectively.

Following a 24 h fasting period at the end of the feeding trial, large yellow croakers were euthanized (MS-222 at 10 mg L^−1^), counted and weight from each cage to determine the survival rate (SR), final body weight (FBW), weight gain (WG), specific growth rate (SGR), feed efficiency (FE), and protein efficiency ratio (PER). A total of 10 fish per cage were randomly selected for sampling, 4 fish of which were used to determine morphological parameters including condition factor (CF), viscerosomatic index (VSI), and hepatosomatic index (HSI). Then, the proximal intestine (PI), middle intestine (MI), and distal intestine (DI) from the 4 same fish was stored in 10% neutral formaldehyde for the analysis of morphology. The liver and intestine samples from 6 selected fish per cage were frozen immediately in liquid nitrogen and stored at -80°C. The livers were collected for the enzyme activities and genes expression analysis. The intestine samples were then sent to Majorbio Bio-Pharm Technology Co., Ltd. (Shanghai, China) for 16S rDNA-based microbiota analysis.

### 2.2. Amino Acids Profile

A total of 0.1 g diets with 10 mL of 6 N HCL was digested at 110°C in glass tubes sealed with a nitrogen atmosphere for 24 h. The solution was filtered and flash evaporated to remove any acid. The acid free sample was further made up with 0.05 N HCL and filtered using a 0.2 *μ*m nylon membrane filter (Whatman, Whatman plc, Kent, UK) to remove any residue. The L-8900 amino acid analyzer (Hitachi, Japan) was approached to determine the amino acids profiles of the diets.

### 2.3. Intestinal Histology

Fresh intestine tissue was fixed with 4% paraformaldehyde before paraffin sections were prepared (Servicebio, Hangzhou, China). Briefly, after fixation for at least 24 h, tissue samples were trimmed appropriately in a fume hood before being dehydrated in ethanol with concentration increasing incrementally from 75% to 100%. Intestine samples were then embedded in paraffin and sliced into sections of 4 *μ*m using a microtome (HistoCore AUTOCUT, Leica, Germany). They were stained with haematoxylin and eosin (H&E) [23], and images were acquired under a microscope (Nikon Eclipse CI, Tokyo, Japan). Mucosal thickness (MT), villi height (VH), and villi width (VW) were measured with ImagePro Plus6.0 software.

### 2.4. Liver Enzyme Activity Analysis

The liver samples were homogenized on ice in 9 volumes (*w* : *v*) of ice cold physiological saline 8.9 g/mL, then centrifugated at 3000 rpm for 10 min at 4°C (Eppendorf centrifuge 5810R, Germany) to collect the supernatant for enzyme activity analysis. The activities of the liver alanine and aspartate aminotransferase (ALT and AST) acid- and alka-line phosphatase (ACP and AKP) were evaluated using commercial assay kits (Jiancheng Bioengineering Institute., Nanjing, China) according a described method [24]. The enzyme specific activity was expressed as unit/g soluble protein. The protein concentration of hepatic homogenates was determined using a protein assay kit (Jiancheng Bioengineering Institute, Nanjing, China).

### 2.5. Real-Time Quantitative PCR (RT-qPCR) Analysis of Liver Health Related Genes

The total RNA of liver was extracted with an Eastep® RNA extraction kit (LS1040, Promega Biotech, China) according to the manufacturer's instruction. The quality of RNA was assessed by a 1.2% agarose gel electrophoresis, and the quantity of total RNA was measured with a spectrophotometer (NP80, IMPLEN, Germany). First- strand cDNA synthesis was performed by using an Eastep® RT Master Mix Kit (LS2050, Promega Biotech, China). *β*-Actin was used as reference gene after the stability of its expression was confirmed. The specific primers for alkaline phosphatase (*akp*), acid phosphatase (*acp*), alanine aminotransferase (*alt*), and aspartate aminotransferase (*ast*) were designed by NCBI online tools (Supplementary Table 1). RT-qPCR was performed in a 20 *μ*L reaction volume including 10 *μ*L SYBR Green premix, 0.8 *μ*L cDNA template, 0.4 *μ*L forward and reverse primers (10 *μ*M), and 8.4 *μ*L diethyl pyrocarbonate-treated water. The reaction conditions are as follows: 95°C 30 s, 40 cycles of 95°C 6 s, and 60°C 25 s. After obtaining data, the 2^−*ΔΔ*Ct^ method was employed to calculate gene expression level [25] and then subjected to statistical analysis.

### 2.6. DNA Extraction and PCR Amplification

The E.Z.N.A. Soil DNA Kit (Omega Bio-tek Inc., Norcross, USA) was used to extract total DNA from each intestinal sample according to the manufacturer's instructions. A Nano Drop 2000 UV–vis spectrophotometer (Thermo Scientific, Wilmington, USA) was used to assess the final DNA concentration and purification, and DNA quality was evaluated using a 1% agarose gel electrophoresis. With bacterial primers 338F 5′-ACTCCTACGGGAGGCAGCAG-3′ and 806R 5′-GGACTACHVGGGTWTCTAAT-3′, the V3-V4 hypervariable portions of the 16S rRNA gene were amplified. PCR amplification was performed as follows: 95°C for 3 min, 27 cycles of 95°C for 30 s, 55°C for 30 s, and 72°C for 45 s, and 72°C for 10 min 4°C using a Thermocycler PCR system (Gene Amp 9700, ABI, USA). The PCR mixtures contains 4 *μ*L of 5 × *TransStart* FastPfu buffer, 2 *μ*L of 2.5 mM dNTPs, 0.8 *μ*L of each primer (5 *μ*M), 0.4 *μ*L of *TransStart* FastPfu DNA Polymerase, and 10 ng of DHA template and diethyl pyrocarbonate-treated water (to 20 *μ*L). The resulting PCR products of bacteria were extracted from a 2% agarose gel, further purified with the AxyPrep DNA Gel Extraction Kit (Axygen Biosciences, USA), and quantified with QuantiFluor-ST (Promega, USA) according to the manufacturer's protocol.

### 2.7. Illumina MiSeq Sequencing and Data Processing

Majorbio Bio-Pharm Technology Co., Ltd. filtered and pooled the bacteria and products in equimolar levels before paired-end sequencing (2 × 300) using an Illumina MiSeq platform (Illumina, San Diego, USA) according to normal protocols (Shanghai, China). Trimmomatic quality-filtered and FLASH merged raw fastq files of bacterial reads received from MiSeq sequencing [26, 27]. The UPARSE (version 7.0) was used to cluster the processed sequences into operational taxonomic units (OTUs) with a minimum of 97% [28]. The RDP Classifier method was used to compare the taxonomy of the bacterial sequence to the SILVA database (138/16S-bacteria) with a 70% confidence level. Phylogenetic Investigation of Communities by Reconstruction of Unobserved States was used to anticipate potential functional changes in the bacterial community in intestinal samples (PICRUSt) [29].

The alpha-diversity.py in Quantitative Insights into Ecology (QIIME, Version 1.9.1) was used to calculate the alpha diversity of intestinal bacteria. The bacterial communities in the samples were visualized using vegan package in R3.3.1 through nonmetric multidimensional scaling (NMDS) based on the Bray-Curtis distance. The linear discriminate analysis (LDA) effect size (LEfSe) method was approached to analyze the high-dimensional microbial taxa [30].

### 2.8. Statistical Analysis

Statistical analysis was performed using SPSS 22.0 (SPSS, Inc., Chicago, USA). Data were tested for normal distribution and homogeneity of variance via the Kolmogorov–Smirnov test and Levene's test. Differences in the physicochemical profiles of intestine samples were tested by one-way analysis of variance (ANOVA) with Tukey's test; *P* < 0.05 was considered a statistically significant difference. Additionally, orthogonal polynomial contrasts were used to assess the significance of linear or quadratic models, to describe the response of the dependent variable to dietary FM replacement levels. The results of growth performance, intestinal histology, liver enzyme activities, and genes expression were presented as means ± SD (*n* = 4). The intestinal microbiota data were presented as means ± SD (*n* = 3).

## 3. Results

**Figure 1:**
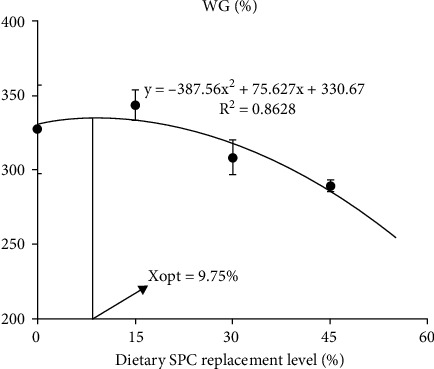
The relationship between the dietary SPC replacement levels and the WG of large yellow croaker fed the experimental diets. The Xopt represents the optimal dietary SPC protein levels for the maximum WG of large yellow croaker. WG: weight gain.

As shown in Table 3, the SR, HSI, VSI, and CF of large yellow croakers were no affected by the dietary SPC inclusion levels (*P* > 0.05). However, the FBW, WG, and SGR of fish fed SPC30 were not different from those fed other diets (*P* > 0.05). Compared to fish fed SPC45, these fed FM and SPC15 had higher FBW, WG, and SGR (*P* < 0.05). The FE and PER were significantly higher in fish fed SPC15 than those fed SPC30 and SPC45 (*P* < 0.05), while no difference was observed between FM and SPC15 group (*P* > 0.05). Regression analyses on FBW, WG, SGR, and FE showed the significant negative linear and quadratic responses to increasing dietary SPC inclusion levels (*P* < 0.05). Based on the quadratic response of WG to different diets, the best growth performance was observed when the replacement of FM with SPC was 9.75% (Figure 1).

**Figure 2:**
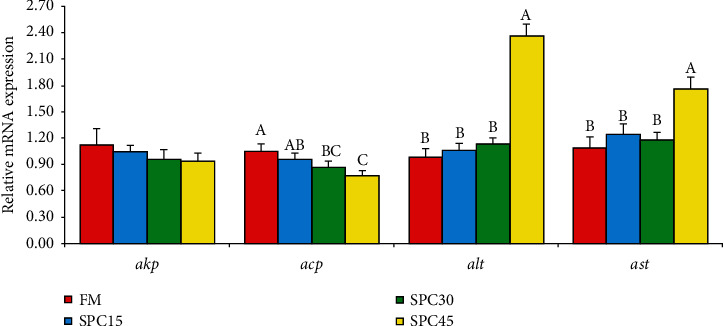
The liver health related gene expression levels in the liver of large yellow croaker fed the experimental diets. *Akp*: alkaline phosphatase; *acp*: acid phosphatase; *alt*: alanine aminotransferase; *ast*: aspartate aminotransferase.

Table 4 showed that the AKP and AST activities in the liver of large yellow croakers were not influenced by dietary FM replacement levels with SPC (*P* > 0.05). The ACP activity and decreased significantly (*P* < 0.05) and showed negative linear and quadratic responses to increasing dietary SPC inclusion levels (*P* < 0.05). The trend of ALT activity was totally opposite (*P* < 0.05). The mRNA expression of *alt* and *ast* in FM, SPC15, and SPC30 were similar and significantly lower than that in SPC45 (*P* < 0.05) (Figure 2). The *acp* expression level showed a significantly decrease trend with dietary SPC levels increasing from 0 to 45% (*P* < 0.05).

**Figure 3:**
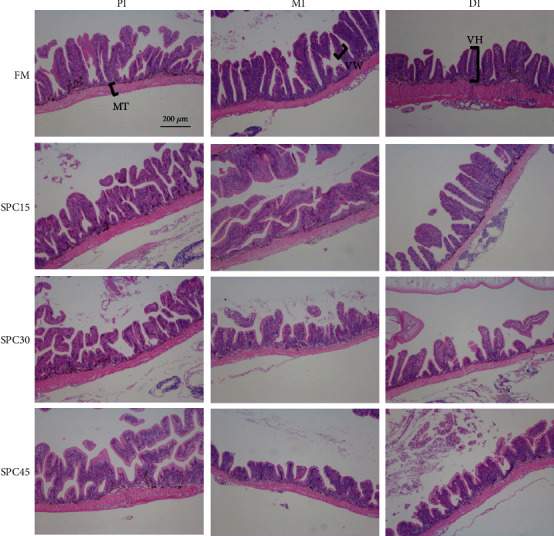
The intestine morphology of large yellow croakers fed the experimental diets. PI: proximal intestine; MI: middle intestine; distal intestine; VH: villi height; VW: villi length; MT: muscular thickness.

The intestinal morphometry of large yellow croaker fed the experimental diets was displayed in Table 5 and Figure 3. The VW and MT in PI, MI and DI were not influenced by the dietary FM replacement level with SPC (*P* > 0.05). The VH in PI of fish fed FM, and MI of fish fed FM and SPC15 were significantly higher than those fed SPC30 and SPC45 (*P* < 0.05). However, there was no difference between the VH of PI in SPC15 and other groups (*P* > 0.05). Fish fed diet SPC15 had significantly higher VH in DI than those fed diet SPC45 (*P* < 0.05). However, the VH of DI in FM and SPC30 was not different to SPC15 and/or SPC45. The VH in PI, MI and DI showed negative linear and quadratic responses to increasing dietary SPC inclusion levels (*P* < 0.05).

In total, 962 OTUs assigned into 33 different phyla, 302 families, or 499 genera were identified in the intestine samples (Supplementary Table 2). The rarefaction curves tended toward the saturation plateau (Supplementary Figure 1), meanwhile the Good's coverage index revealed that the amounts of obtained bacterial species in five different groups were>99% (Table 6). The coverage of fish fed diet SPC30 was significantly lower than those fed FM and SPC15 (*P* < 0.05). The values of in Sobs, Shannon, Ace, and Chao showed a significantly quadratic response to dietary SPC inclusion levels and were highest in SPC15 group (*P* < 0.05). While the Simpson was lowest in SPC15 group, there was no difference among the alpha diversity of FM, SPC30, and SPC45 group (*P* > 0.05).

**Figure 4:**
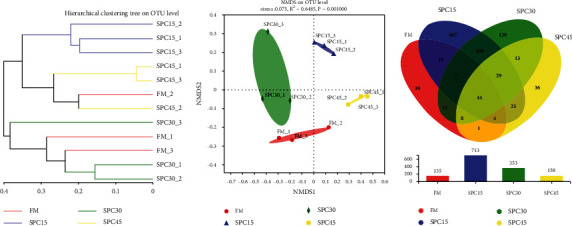
The intestinal microbial structure of large yellow croakers fed the experimental diets. (a) Multiple samples similarity tree; (b) nonmetric multidimensional scaling (NMDS); (c) Venn diagram.

NMDS was conducted to determine the *β* diversity to analyze the extent of similarities in microbial communities of fish in different groups (Figure 4(b)). The further the components were separated, the greater the difference. The NMDS plot showed that the components of fish in the different experimental groups were clustered and separated from each other. The gut microbiota structure from FM, SPC30, and SPC45 group were similar and clustered within one higher branch, whereas was distinct from SPC15 group and formed another branch (Figure 4(a)). A Venn diagram showed that 44 OTUs were shared by the four different groups (Figure 4(c)). The unique OTUs in FM, SPC15, SPC30, and SPC45 were 39, 487, 139, and 36, respectively. 135, 713, 353, and 156 were the total OTUs in the four groups.

**Figure 5:**
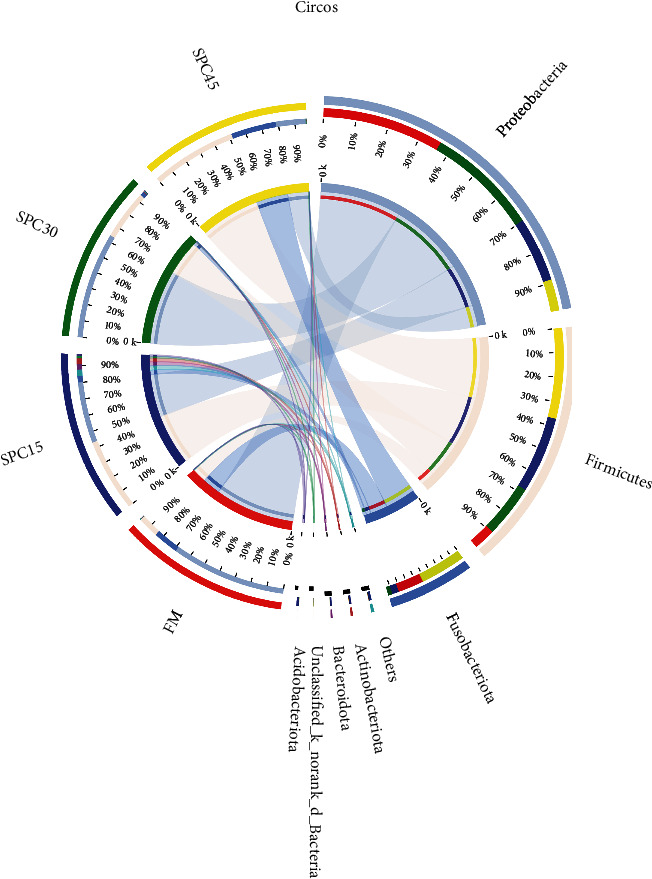
Differences in relative abundance of microbial community in intestine of large yellow croaker fed the experimental diets on phylum level.

Bacterial community dynamics of gut microbiota in fish fed different experimental diets were shown in Figure 5. At the phylum level, *Proteobacteria*, *Firmicutes*, and *Fusobacteriota* were the predominant bacterial in the intestine of fish among all groups (Figure 5). The dominant phyla in FM group were *Proteobacteria* (71%), *Fusobacteriota* (16%), and *Firmicutes* (13%) while the SPC15 group was dominated by *Firmicutes* (45%), *Proteobacteria* (38%), and *Fusobacteriota* (4.1%). The relative abundance of bacterial phyla in SPC30 group was *Proteobacteria* (65%), *Firmicutes* (31%), and *Fusobacteriota* (2.5%) while in SPC45 group, *Firmicutes* (53%), *Fusobacteriota* (28%), and *Proteobacteria* (18%).

**Figure 6:**
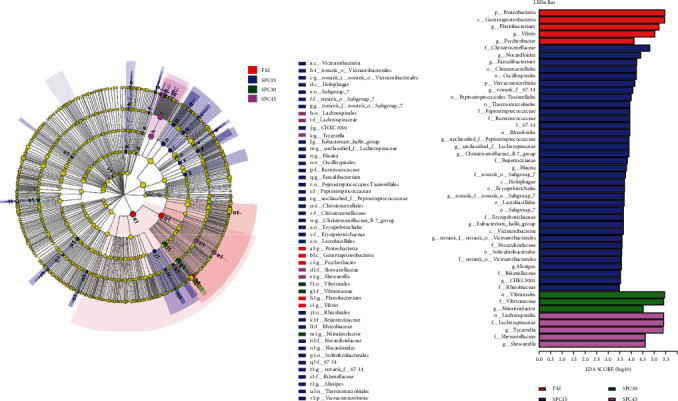
LEfSe analysis identified the most differentially abundant taxons between large yellow croakers fed different experimental diets. (a) Cladogram was calculated by LefSe. (b) Histogram of linear discriminant analysis (LDA) scores for differentially abundant taxon. Differences were presented by the color of the most abundant taxa (red, blue, green, and purple indicated one taxon with significantly higher relative abundance in FM, SPC15, SPC30, and SPC45 treatments, respectively).

LEfSe analysis was performed to determine the difference in gut microbial community composition of fish fed FM, SPC15, SPC30, and SPC45 diets (Figures 6(a) and 6(b)). In the FM group, phylum *Proteobacteria*, class *Gamma-proteobacteria* (class) and genus *Photobacterium*, *Psychrobacter*, and *vibrio* were significantly enriched, *Vibrionales* (from order to family) and genus *Nitratireductor* were enriched in SPC30 group, while *shewanellaceae* (from family to genus), *Lachnospiraceae* (from order to family), and genus *tyzzerella* were significantly enriched in the SPC 45 group. More bacteria were significantly enriched in the SPC15 group, including the phylum *Verrucomicrobiota*, class *Vicinamibacteria*, and no-rank *Vicinamibacteria* (from family to genus), class *holophagae* and *Subgoup* 7 (from order to genus), order *Oscillospirales* and its family *Ruminoccoccaceae* (family and its genus *faecalibacterium*), *Peptostreptoccoccale* (from order to genus), *Christensenellales* (from order to genus), *Erysipelotrichales* (from order to family), order *Lactobacillales*, *Thermomicrobiales* and *Rhizobialaes* (its families *Rhizobiaceae* and *Beijerinckiaceae*), *Nocardioidaceae* (from family to genus), order *Solirubrobacterales* and its family *67-14* (from family to genus), family *Rikenellaceae,* and its genus *Alistipes*, genus CHKCI001, *Eubacterium_hallii_group*, unclassified *Lachnospiraceae*, *Blautia*.

**Figure 7:**
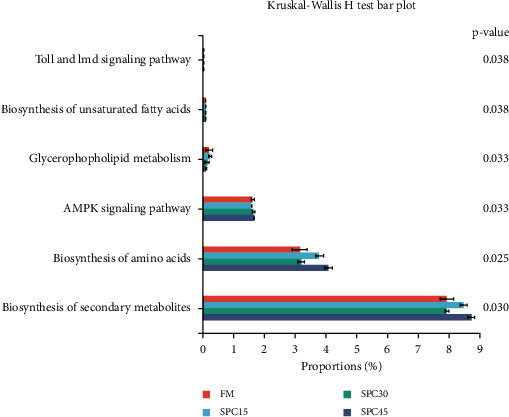
Functional analysis of the microbial composition of large yellow croakers fed the experimental diets. Differences were considered significant at *P* < 0.05.

In Figure 7, significant differences were observed in the KEGG pathways among the five treatments. Biosynthesis of unsaturated fatty acids, PI3K-Akt signaling pathway, AMPK signaling pathway, Glycerophospholipid metabolism, Biosynthesis of secondary metabolites, Biosynthesis of amino acids, Toll, and Imd signaling pathway.

## 4. Discussion

Many studies performed on the replacement of FM showed that SPC could totally replace FM and be good feed ingredient for fish species, such as Atlantic halibut (*Hippoglossus hippoglossus*) [8], rainbow trout [31], and longfin yellowtail (*Seriola rivoliana*) [32]. Study in giant grouper (*Epinephelus lanceolatus*) suggested that at least 40% FM could be replaced by SPC without impairing the growth performance [33]. Moreover, SPC combined with the supplementation of lysine and methionine could replace more than 40% FM in the diet of juvenile starry flounder (*Platichthys stellatus*) at least [34], whereas 100% FM in the diet of large yellow croaker (initial weight 10.50 ± 0.04 g) as no significant effects on growth performance [13]. In the present study, the SR, HSI, VSI, and CF were not affected by dietary SPC levels. The best growth performance was observed when the dietary SPC inclusion was 9.75%, and WG and SGR of fish fed SPC45 were significantly lower than those fed FM and SPC15. These results showed that large yellow croaker could tolerate more than 30% FM replaced by SPC. This is a lower content of SPC than has been shown to be effective in previous study [13]. The discrepancy could be due to different initial weight and the crystal amino acid (methionine and lysine) supplementation, which contributes to the higher tolerance of fish and crustacean as reported in Japanese flounder (*Paralichthys olivaceus*) [35], juvenile cobia (*Rachycentron canadum*) [36], and juvenile swimming crab, *Portunus trituberculatus* [10].

AST and ALT in hepatopancreas are the two most important enzymes in the process of metabolism of AAs and key indicators of cellular damage and liver function [37]. The high levels of transaminases often are the sign of ill health. ACP and AKP are affected by nutritional status, environmental changes, diseases and growth stages, and could reflect the health status of animals [38]. In this study, the activity and mRNA expression of ACP in liver decreased significantly, whereas the activity of ALT and expression of *alt* and *ast* were opposite with increasing dietary SPC levels. Thus, large yellow croaker fed SPC45 maybe in ill health, which agreed with the lower growth performance described above. Similar results were reported in soft-shelled turtle (*Pelodiscus sinensis*) [24], darkbarbel catfish (*pelteobagrus vachelli*) [39], and *P. trituberculatus* [10].

In the current study, the trend of FE and PER in response to dietary SPC inclusion level was consistent with WG and SGR. Therefore, the effects of dietary SPC replacing FM on the growth of large yellow croaker were significantly related to improvements in nutrient digestion and absorption. Intestine is the primary location of nutrient digestion and absorption in fish, and its structural integrity is critical [40]. Morphological indices such as VH, VW, and MT are indictors of intestinal absorptive capacity [41, 42]. A decrease in intestinal VH and quantity often signifies a corresponding decrease in the absorption area of the digestive tract, and, consequently, a reduction in nutrient absorption [43]. In the current study, the VH of PI, MI, and DI were similar in fish fed FM and SPC15. However, fish fed SPC45 had significantly lower VH in PI, MI and DI when compared with those fed diet FM and/or SPC15. This may contribute to the receiving of the inappropriate diets, which could reduce the functional surface area of the intestinal and lead to lower ability of digestion and absorption [44]. Changes in intestinal morphology could possibly explain the lower FE and growth performance metrics of fish fed diets containing 45% SPC. Similar results were found in juvenile hybrid grouper (*E. fuscoguttatus* ♀ × *E. lanceolatus* ♂) fed diets with higher than 55% FM protein being replaced by SPC [45]. Given that the positive correlation between intestine morphology, we analyzed the intestinal microbiota to further investigate the response of large yellow croaker to dietary SPC contents.

Intestinal microbiota modulates host physiological processes and plays a critical role in promoting and maintaining the health of the host. It was reported that the intestinal microbiota of fish was not only closely related to the host but also considerably influenced by the surrounding environment, including water and diets [46–48]. In the present study, the Sobs, Shannon, Ace, and Chao were highest in SPC15 group, while the Simpson was lowest in SPC15 group. No difference was observed among the alpha diversity of FM, SPC30, and SPC45 group. The positive effects in bacterial diversity were also observed in Atlantic salmon (*Salmo salar*) fed diets containing 5% SPC [49], totoaba (*Totoaba macdonaldi*) fed diets containing 45% SPC [50], and rainbow trout (*Oncorhynchus mykiss*) fed diets containing 25% soybean meal [9]. It was reported in adult humans and mammals that a diverse microbiota has frequently been linked with a balanced, well-functioning metabolism [51, 52]. Therefore, our finding indicated that the 15% SPC inclusion could enhance nutrient uptake and metabolism in juvenile large yellow croaker.

In the present study, *Proteobacteria*, *Firmicutes*, and *Fusobacteriota* were the predominant bacterial phyla in the intestine of fish regardless of the dietary SPC inclusion level. *Proteobacteria*, *Fusobacterium*, and *Firmicutes* were also dominant in many other fish species and considered as members of the “core gut microbiota” [53–55]. The existence of similar bacterial taxa in the gut microbiota of a variety of fish species suggests that these bacteria are involved in important host gut functions like digestion, nutrition absorption, and immune response [53]. Interestingly, recent study on *L. crocea* reported that *Fusobacteriota* was only identified in the intestinal microbiota of 1-year-old instead of 12-day-old fish [56]. Thus, fish developmental stage may contribute to these different results. The ability of bacteria of the *Fusobacteria* phylum to synthesis vitamins and excrete butyrate is well recognized, and this is important since butyrate has numerous beneficial impacts on the health of the intestinal tract and peripheral tissues in vertebrates [57–59]. To investigate the response of intestinal microbiota to the replacement level of FM, dominant colony was identified subsequently in large yellow croaker fed diets with different SPC contents consequently.

Lactic acid bacteria within order *Lactobacillales* are among the most used probiotics in aquaculture because these bacteria reproduce rapidly; produce antimicrobial compounds such as organic acids, lactic acid, bacteriocins, and hydrogen peroxide; and stimulate nonspecific immune responses in hosts [60]. Previous study demonstrated that *L. vannamei* fed the *Lactobacillus* spp. diet exhibited higher WG and survival, lower abundance of pathogenic bacterial load (e.g., *Vibrio*) than the shrimp fed control diet (without *Lactobacillus* spp. addition) [61]. The mixture of lactic acid bacteria in diet led to increasing VH and the abundance of *Rhizobium*, which were considered to be beneficial effects in Nile tilapia (*Oreochromis niloticus*) [62]. Atlantic salmon (*Salmo salar* L) fed SPC diet showed a higher abundance of family *Lactobacillaceae* in proximal intestine and similar growth performance than those fed FM diet [63]. In the study, the enrichment of order *Lactobacillales* and order *Rhizobiaceae* (including family *Beijerinckiaceae* and *Rhizobiaceae*) in fish fed SPC15 could indicating the beneficial effects of optimal dietary SPC inclusion. However, genus *Peptostreptococcaceae* was observed to be higher abundance in SPC15 other treatments. Similar trend of genus *Lactobacillus* and *Peptostreptococcaceae* abundance was also observed in *Coilia nasus* get infected by *Acanthosentis cheni* [64]. *Peptostreptococcaceae* is a group of obligate anaerobe bacteria and is heritable across humans, mice and fish, cooccur, and inhabit the small intestine [65]. The high abundance of *Peptostreptococcaceae* occurred in human get Crohn's disease (subtype of inflammatory bowel disease) and rectal cancer patients [66, 67]. Moreover, the relative abundance of *Peptostreptococcaceae* was opposite to beneficial microbiomes such as *Enterococcus*, *Bacteroides*, and *Prevotella* in mice [68]. Thus, *Peptostreptococcaceae* may also act as pathogen in the intestine of large yellow croaker. Beside order *Lactobacillales* and *Peptostreptococcaceae* (from order to genus), a quantity of bacteria belonging to phylum *Firmicutes* (e.g. class *Vicinamibacteria* and *Christensenellales*, order *Erysipelotrichales* and family *Nocardioidaceae*) was enriched in intestine of large yellow croaker fed SPC15. Although information on the function of about each genus in fish is limit, Phylum *Firmicutes* was reported to possess the ability to improve the digestibility and immune status of fish to counteract the effects of pathogenic bacteria [69]. These indicated that SPC replace 15% FM could improve the health status of large yellow croakers.

It was reported that *Vibrio* and *Photobacterium* are commonly found in carnivores [70]. *Photobacterium* act as mutualistic bacteria in the host gut aiding with chitin digestion [71]. However, some also produce harmful enzymes such as neuraminidases [72]. It was demonstrated that *Photobacterium* in intestine of *L. crocea* was relatively close to *Photobacterium damselae* at the evolutionary level and was associated with pathogenicity [73]. *Vibrio* is one of the most important bacterial genera in aquaculture, with both pathogenic and probiotic (health-promoting) species [70, 74]. Several *Vibrio* bacteria such as *V. harveyi, V. sinaloensi* ,and *V. orientalis* are generally disease related [75, 76]. Recent study on the gut microbiota of *L. crocea* revealed that fish fed commercial feed showed higher *Vibrio* abundance than those fed fresh feed [73]. In terms of pathogenesis associated with high plant protein diets, increased relative abundance of *Psychrobacter* sp. has been proposed [49]. However, the abundance of *Psychrobacter* sp. could inhibit pathogenic *V. harveyi, V. metschnikovi*, and *V. alginolyticus* in juvenile grouper *E. coioides* [77]*. Shewanella* belongs to family *shewanellaceae*, was reported as opportunistic pathogens exist in Pacific white shrimp (*L Vannamei*) [78], but as probiotics in several marine fish [70]. Moreover, *Tyzzerella* has been characterized as a genus that predisposes hosts to diarrhea [79]. Moreover, a higher abundance of *Tyzzerella* is correlated with a higher risk of cardiovascular disease [80] and has been associated with dietary quality in human [81]. In the present study, genus *Photobacterium*, *Psychrobacter*, and *vibrio* belonged to class *Gamma-proteobacteria* within phylum *Proteobacteria* were significantly enriched in fish fed FM diet*. Vibrionaceae* family and *Vibrionales* order were enriched in fish fed SPC30 diet. *Tyzzerella* belongs to the *Lachnospiraceae* family within the phylum *Firmicutes*, genus *Shewanella*, and family *shewanellaceae* within class *Gamma-proteobacteria* and phylum *Proteobacteria* were enriched in large yellow croaker fed SPC45 diet. However, compared to fish fed SPC15 diet, those fed other diets had higher abundance of bacteria belonged to phylum *Proteobacteria,* which was regard as the most stable bacteria in *L. vannamei* and the abundance of *Proteobacteria* was not affected by environment or diets [78]. Thus, the abundance of these bacteria is either low or at a certain level that maintains a balance with the number of other microbes in large yellow croaker. Whereas, the higher abundance of *Tyzzerella* in large yellow croaker fed diet SPC45 could be the response to lower quality diet caused by SPC replacing 45% dietary FM protein, which agreed to the growth performance. *Tyzzerella* could be the bacteria indicator of intestinal in large yellow croaker fed high SPC content diet.

It has been demonstrated that alternative protein sources can alter the gut microbiome of the host to have a beneficial impact on growth and immunity [82]. In this study, dietary fish oil content increased with increasing dietary SPC inclusion levels due to its lower lipid content than FM, which was also considered to contain relative balanced amino acids profile. Fish oil is richness in the content of long chain polyunsaturated fatty acids, which prefer to deposit in phospholipids. Thus, these vary gut microbiota communities and the enrichment of Toll and Imd signaling pathway, Biosynthesis of unsaturated fatty acids, Glycerophospholipid metabolism, PI3K-Akt signaling pathway, AMPK signaling pathway, Biosynthesis of secondary metabolites, and Biosynthesis of amino acids pathway indicated the regulation of immunity capacity and an acceleration of lipid and protein metabolism, which could be the adjustment of absorbing more SPC in *L. crocea* and need to be further studied.

## 5. Conclusions

Under this experimental condition, SPC could be good ingredient for large yellow croaker as the growth performance, feed utilization, morphological parameter, and liver health were not affected, the intestinal microbiota communities were well regulated when 15% FM was replaced. Higher FM replacement level than 30% would lead to a decrease in health status, VH, and an addition in the abundance of pathogen such as *Vibrio* and *Tyzzerella*, and then the reduction of WG, SGR, and FE. *Tyzzerella* could be the bacteria indicator of intestinal in large yellow croaker fed high SPC content diet. Based on the quadratic regression analysis of WG, the best growth performance could be observed when the replacement of FM with SPC was 9.75%. Moreover, the substitution of FM by SPC accelerated the metabolism of lipid, protein, and immunomodulating based on pathway enrichment of the bacterial abundance.

## Figures and Tables

**Table 1:** Formulation and proximate composition of the experimental diets (dry matter).

Ingredients^a^ (%)	Diets			
	FM	SPC25	SPC30	SPC45
Fish meal	40.00	34.00	28.00	22.00
Soybean protein concentrate	0.00	6.02	12.03	18.04
Soybean meal	21.12	21.12	21.12	21.12
Wheat flour	15.00	15.00	15.00	15.00
Fish oil	2.57	2.75	2.93	3.11
Soybean oil	2.57	2.75	2.93	3.11
Soybean lecithin	1.50	1.50	1.50	1.50
Ca(H_2_PO_4_)_2_	1.50	1.50	1.50	1.50
Choline chloride	0.30	0.30	0.30	0.30
Mineral premix^b^	2.00	2.00	2.00	2.00
Vitamin premix^b^	3.00	3.00	3.00	3.00
Cellulose	10.44	10.06	9.69	9.32
Total	100.00	100.00	100.00	100.00
Nutrition level^c^				
Dry matter	88.66	89.52	87.32	88.95
Crude protein	44.50	44.11	43.48	44.55
Crude lipid	9.64	9.77	9.45	9.69
Ash	5.46	4.98	4.38	4.57

^a^All ingredients purchased from Fuzhou Haima Feed Co., Ltd., Fuzhou 350002, China. ^b^Vitamin premix and mineral premix were based on Wei et al. [20]. ^c^Means of two replicate analyses per sample.

**Table 2:** The amino acids composition of the experimental diets (dry matter).

Amino acid	FM	SPC15	SPC30	SPC45
EAA^a^				
Thr	1.83	1.81	1.76	1.77
Val	1.90	1.89	1.86	1.85
Met	0.92	0.84	0.80	0.72
Ile	1.79	1.75	1.67	1.69
Leu	3.27	3.17	3.07	3.09
Phe	2.20	2.07	1.96	2.02
Lys	2.68	2.70	2.73	2.62
His	1.14	1.13	1.19	1.11
Arg	2.98	2.82	2.65	2.72
NEAA^b^				
Asp	4.99	4.77	4.46	4.65
Ser	2.31	2.16	2.03	2.12
Glu	8.63	8.70	8.10	8.54
Gly	1.80	1.85	1.87	1.80
Ala	2.06	2.13	2.18	2.08
Cys	0.49	0.49	0.41	0.43
Tyr	1.56	1.52	1.38	1.43
Pro	2.85	2.66	2.46	2.59
EAA	18.71	18.19	17.69	17.92
NEAA	24.68	24.30	22.90	23.64
TAA ^c^	43.39	42.48	40.58	41.56

^a^EAA: the essential amino acid. ^b^NEAA: nonessential amino acid. ^c^TAA: total amino acid.

**Table 3:** Growth performance, feed utilization and morphological parameters of large yellow croakers fed the experimental diets.

Items	FM	SPC15	SPC30	SPC45	Linear		Quadratic	
					*P* value	*R* ^2^	*P* value	*R* ^2^
IBW^a^ (g)	20.23 ± 1.06	19.29 ± 1.73	20.05 ± 2.43	18.45 ± 0.65				
FBW^b^ (g)	87.20 ± 8.21^a^	85.59 ± 8.67^a^	81.93 ± 10.61^ab^	71.88 ± 2.75^b^	0.013	0.363	0.030	0.416
WG^c^ (%)	327.33 ± 29.64^a^	343.31 ± 10.23^a^	308.46 ± 11.75^ab^	289.56 ± 3.70^b^	0.003	0.481	0.004	0.577
SGR^d^ (%/d)	2.59 ± 0.12^a^	2.66 ± 0.04^a^	2.51 ± 0.05^ab^	2.43 ± 0.02^b^	0.003	0.487	0.003	0.590
FE^e^	0.64 ± 0.02^ab^	0.71 ± 0.04^a^	0.62 ± 0.05^b^	0.59 ± 0.02^b^	0.015	0.356	0.006	0.540
PER^f^	1.47 ± 0.07^ab^	1.60 ± 0.08^a^	1.43 ± 0.11^b^	1.31 ± 0.05^b^	0.021	0.324	0.003	0.324
SR^g^ (%)	73.64 ± 7.55	78.89 ± 7.96	73.05 ± 5.31	75.35 ± 7.39	0.965	≤0.001	0.920	0.013
HSI^h^ (%)	1.85 ± 0.14	1.6 ± 0.11	1.77 ± 0.14	1.73 ± 0.10	0.536	0.028	0.303	0.168
VSI^i^ (%)	7.13 ± 0.51	6.87 ± 0.7	6.41 ± 0.38	6.73 ± 0.59	0.195	0.117	0.256	0.189
CF^j^ (g/cm^3^)	1.07 ± 0.08	1.11 ± 0.05	1.04 ± 0.03	1.09 ± 0.06	0.914	≤0.001	0.994	≤0.001

Data in the table are presented as means ± SEM (*n* = 4). Values in the same raw with different superscripts are significantly different (*P* <0.05). ^a^IBW: initial body weight; ^b^FBW: final body weight; ^c^WG: weight gain (%) = 100 × (FBW (g) − IBW (g))/FBW; ^d^SGR: specific growth rate (%/d) = 100 × (ln(FBW (g)) − ln(IBW (g)))/days; ^e^FE: feed efficiency = (FBW (g) − IBW (g))/feed intake (g, dry weight); ^f^PER: Protein efficiency ratio = weight gain (g, wet weight)/protein intake (g, dry weight); ^g^SR: survival rate (%) = 100 × (final amount of fish/initial amount of fish); ^h^HSI: hepatosomatic index (%) = 100 × (liver weight (g/fish)/body weight (g/fish)); ^i^VSI: viscerosomatic index = 100 × viscera weight (g)/final body (g); ^j^CF: condition factor = 100 × FBW (g/fish)/(body length (cm/fish))^3^.

**Table 4:** The liver enzyme activities of large yellow croakers fed the experimental diets.

Items	FM	SPC15	SPC30	SPC45	Linear		Quadratic	
					*P* value	*R* ^2^	*P* value	*R* ^2^
AKP^a^ (U/gprot)	31.35 ± 3.37	28.19 ± 4.27	33.95 ± 6.23	30.14 ± 2.33	0.837	0.003	0.971	0.005
ACP^b^ (U/gprot)	88.47 ± 6.25^a^	83.04 ± 7.48^ab^	76.81 ± 4.58^ab^	70.13 ± 6.64^b^	≤0.001	0.609	0.002	0.610
ALT^c^ (U/gprot)	327.99 ± 22.50^b^	350.21 ± 19.35^ab^	368.81 ± 24.92^ab^	384.11 ± 19.69^a^	≤0.001	0.550	0.005	0.554
AST^d^ (U/gprot)	213.15 ± 25.37	240.19 ± 32.44	246.65 ± 20.37	262.21 ± 35.65	0.025	0.311	0.082	0.320

Data in the table are presented as means ± SEM (n =4). Values in the same raw with different superscripts are significantly different (*P* < 0.05). ^a^AKP: alkaline phosphatase; ^b^ACP: acid phosphatase; ^c^ ALT: alanine aminotransferase; ^d^ AST: aspartate aminotransferase.

**Table 5:** The intestinal morphology of large yellow croakers fed the experimental diets.

Items		FM	SPC15	SPC30	SPC45	Linear		Quadratic	
						*P* value	*R* ^2^	*P* value	*R* ^2^
PI^a^	VH^d^ (*μ*m)	384.56 ± 25.67^a^	366.32 ± 31.27^ab^	323.45 ± 21.66^b^	321.14 ± 16.33^b^	≤0.001	0.569	0.003	0.583
	VW^e^ (*μ*m)	44.56 ± 7.21	41.58 ± 6.87	37.52 ± 7.74	46.37 ± 8.46	0.938	≤0.001	0.319	0.161
	MT^f^ (*μ*m)	71.37 ± 4.33	68.87 ± 5.62	67.32 ± 5.91	73.53 ± 5.15	0.692	0.012	0.254	0.190

MI^b^	VH (*μ*m)	416.33 ± 24.55^a^	364.19 ± 28.77^a^	183.36 ± 28.77^b^	145.02 ± 36.78^b^	≤0.001	0.885	≤0.001	0.886
	VW (*μ*m)	50.35 ± 5.11	54.73 ± 6.64	47.19 ± 7.33	42.79 ± 6.56	0.062	0.226	0.080	0.322
	MT (*μ*m)	58.51 ± 4.87	66.88 ± 6.09	55.43 ± 7.37	57.94 ± 8.22	0.452	0.041	0.574	0.082

DI^c^	VH (*μ*m)	232.35 ± 17.66^ab^	295.71 ± 21.11^a^	237.78 ± 39.22^ab^	195.82 ± 30.79^b^	0.039	0.271	0.004	0.566
	VW (*μ*m)	76.41 ± 18.52	67.47 ± 15.94	68.55 ± 16.44	72.46 ± 17.14	0.730	0.009	0.681	0.057
	MT (*μ*m)	77.51 ± 8.20	67.83 ± 11.49	70.85 ± 13.48	65.87 ± 7.42	0.178	0.126	0.377	0.139

Data in the table are presented as means ± SEM (*n* = 4). Values in the same raw with different superscripts are significantly different (*P* < 0.05).^a^PI: proximal intestine; ^b^MI: middle intestine; ^c^distal intestine; ^d^VH: villi height; ^e^VW: villi width; ^f^MT: mucosal thickness.

**Table 6:** Alpha diversity index of intestinal microbiota of large yellow croaker fed the experimental diets.

Treatments	FM	SPC15	SPC30	SPC45	Linear		Quadratic	
					*P* value	*R* ^2^	*P* value	*R* ^2^
Sobs	65.67 ± 20.11^b^	325.67 ± 114.69^a^	151.67 ± 84.67^ab^	74.67 ± 33.38^b^	0.673	0.019	0.041	0.507
Shannon	1.59 ± 0.43^b^	3.82 ± 0.15^a^	1.86 ± 0.16^b^	1.59 ± 0.16^b^	0.475	0.052	0.052	0.481
Simpson	0.31 ± 0.14^a^	0.07 ± 0.01^b^	0.29 ± 0.09^b^	0.32 ± 0.06^a^	0.489	0.049	0.159	0.335
Ace	69.27 ± 17.46^b^	328.04 ± 115.57^a^	166.94 ± 74.81^ab^	88.49 ± 28.17^b^	0.760	0.010	0.034	0.527
Chao	71.22 ± 17.42^b^	329.07 ± 116.19^a^	163.24 ± 79.90^ab^	89.54 ± 28.07^b^	0.745	0.011	0.041	0.507
Coverage	0.9998 ± 0.0001^a^	0.9998 ± 0.0001^a^	0.9994 ± 0.0001^b^	0.9995 ± 0.0001^ab^	0.034	0.376	0.095	0.407

Data in the table are presented as means ± SEM (*n* = 3). Values in the same raw with different superscripts are significantly different (*P* < 0.05).

## Data Availability

The data that support the findings of this study are available from the corresponding author upon reasonable request.

## Supplementary Materials

Supplementary Materials Supplementary Table 1: sequence of the primers used for q-PCR in this study. Supplementary Table 2: OTUs observed in the experiment. Supplementary Figure 1: rarefaction analysis of the experimental samples. Rarefaction curves of OTUs clustered at 97% phylotype similarity level.
