# Supplementary material for: Partial Substitution of Fish Meal with Soy Protein Concentrate on Growth, Liver Health, Intestinal Morphology, and Microbiota in Juvenile Large Yellow Croaker (Larimichthys crocea)
Source: Aquac Nutr. 2023 Jan 6;2023:3706709. doi: 10.1155/2023/3706709 (PMC9973153; doi:10.1155/2023/3706709)
Supplement: Supplementary Materials — Supplementary Table 1: sequence of the primers used for q-PCR in this study. Supplementary Table 2: OTUs observed in the experiment. Supplementary Figure 1: rarefaction analysis of the experimental samples. Rarefaction curves of OTUs clustered at 97% phylotype similarity level. [file 3706709.f1.docx]

**Supplementary data**

**Title**:

**Partial substitution of fish meal with soy protein concentrate on growth, liver health, intestinal morphology and microbiota in juvenile large yellow croaker (*Larimichthys crocea*)**

Wang et al

Supplementary Table 1. Sequence of the primers used for q-PCR in this study.

| Genes | Primer sequence (5′-3′) | Accession no. |
| --- | --- | --- |
| *akp ^a^* | F: AGGACGAATTGACCACGGAC  R: ACATTCTCCCTTGCACCGTT | HQ113366.1 |
| *acp ^b^* | F: ACAACTCCCGTCATGCAACA  R: TGAGCGTGTTGTCCAAGTCA | XM_010734596.3 |
| *alt ^c^* | F: GACACAGCTCAGATCAGGTCA  R: CCTGTGTATGTCACCCCACG | XM_027291343.1 |
| *ast ^d^* | F: GGCTGAGGCTTTTGGGAACT  R: CGACCGTTGATTGCACTGATG | XM_019255387.2 |
| *β-actin* | F: GACCTGACAGACTACCTCATG  R: AGTTGAAGGTGGTCTCGTGGA | GU584189 |

^a^ AKP, alkaline phosphatase; ^b^ ACP, acid phosphatase; ^c^ ALT, alanine aminotransferase; ^d^ AST, aspartate aminotransferase.

Supplementary Table 2. OTUs observed in the experiment.

| Item | Domain | Kingdom | Phylum | Class | Order | Family | Genus | Species | OTU |
| --- | --- | --- | --- | --- | --- | --- | --- | --- | --- |
| Numbers | 1 | 1 | 33 | 77 | 184 | 302 | 499 | 727 | 962 |


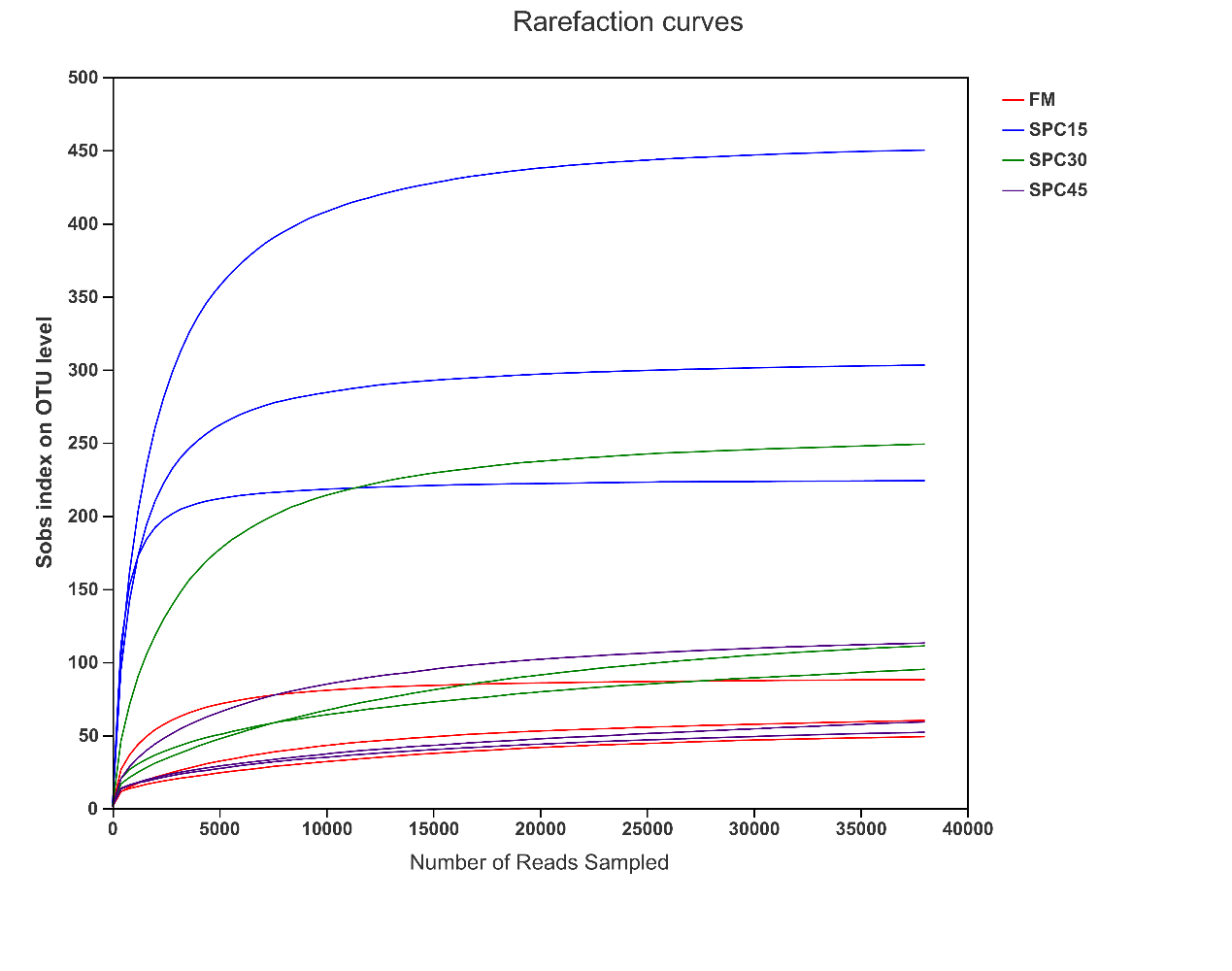


Supplementary Fig 1. Rarefaction analysis of the experimental samples. Rarefaction curves of OTUs clustered at 97% phylotype similarity level.
